# Effect of Elevated Temperature on Physical Activity and Falls in Low-Income Older Adults Using Zero-Inflated Poisson and Graphical Models

**DOI:** 10.3390/info16060442

**Published:** 2025-05-26

**Authors:** Tho Nguyen, Dahee Kim, Yingru Li, Christopher T. Emrich, Jennifer Crook, Ladda Thiamwong, Rui Xie

**Affiliations:** 1 Department of Statistics and Data Science, University of Central Florida, Orlando, FL 32816, USA; 2 College of Nursing, University of Central Florida, Orlando, FL 32826, USA; 3 Department of Sociology, University of Central Florida, Orlando, FL 32816, USA; 4 National Center for Integrated Coastal Research, School of Public Administration, University of Central Florida, Orlando, FL 32816, USA; 5 Disability, Aging and Technology Cluster, University of Central Florida, Orlando, FL 32816, USA

**Keywords:** high temperature, falls, physical activity, older adults, machine learning, mixed undirected graphical models, zero-inflated Poisson regression

## Abstract

High ambient temperature poses a significant public health challenge, particularly for low-income older adults (LOAs) with preexisting health and social issues and disproportionate living conditions, placing them at a vulnerable condition of heat-related illnesses and associated public health risks. This study aims to utilize advanced statistical regression and machine learning methods to analyze complex relationships between elevated temperature, physical activity (PA), sociodemographic factors and fall incidents among LOAs. We collected data from a cohort of 304 LOAs aged 60 and above, living in free-living conditions in low-income communities in Central Florida, USA. Zero-inflated Poisson regression was employed to examine the linear relationships, which reflect the zero-abundant nature of fall incidents. Then, an advanced machine learning approach—the mixed undirected graphical model (MUGM)—was employed to further explore the intricate, nonlinear relationships among daily PA, daily temperature, and fall incidents. The findings suggest that more moderate-to-vigorous PA is significantly associated with fewer fall incidents (RR = 0.90, 95% CI: (0.816, 0.993), *p* = 0.037), after adjusting for other variables. In contrast, elevated temperature is strongly linked to a greater risk of falls (RR = 1.733, 95% CI: (1.581, 1.901), *p* < 0.0001), potentially reflecting seasonal influences. Although higher temperature increases fall events, this effect is mitigated among LOAs with increased sedentary behavior (*p* < 0.0001). Additionally, findings from the MUGM reinforce the intricate nature of falls. Fall counts were highly correlated with race and positively associated with temperature, highlighting the importance of tailoring fall prevention strategies to account for seasonal variations and health disparities, and promoting PA.

## 1. Introduction

### 1.1. Background and Motivation

Rising temperature has become one of the most critical public health concerns in recent years, significantly impacting human health in both direct and indirect ways [1]. Together with other natural and human-made health stressors, extreme heat events are increasingly becoming more frequent and severe, and are worsening health disparities [2]. Whether due to excessively high or low temperatures, these events contribute to an increase in respiratory, cardiovascular, and cerebrovascular diseases, as well as related injuries and mental health illnesses, resulting in more fatalities than all other weather-related disasters combined [3]. Moreover, factors such as population growth, aging, urbanization, and socioeconomic development can either exacerbate or mitigate heat-related risks [3]. Considering current trends in climate change progress and the rapidly growing aging population, heat-related mortality and morbidity are expected to rise [4]. Social, environmental, and economic factors including poverty, racial disparities, limited healthcare access, inadequate education, and unhealthy or unsafe living environments can greatly contribute to health inequities and may have greater impacts on vulnerable populations [5,6]. Among these groups, low-income older adults (LOAs), who bear preexisting health and social issues and disproportionate living conditions, face significant challenges in adaptation abilities or physically responding to extreme weather conditions, placing them at a heightened risk of heat-related illnesses and associated public health risks [7]. Because of their multiple pathophysiological conditions, extreme heat poses a significant risk when engaging in physical activity [8]. The ability to regulate temperature deteriorates due to age-related changes in sweat gland function and blood circulation, making them highly susceptible to heat-related illnesses, such as heat exhaustion and heat stroke, even with mild-to-moderate activity levels [8,9]. Therefore, it is essential for older adults to take extra precautions during hot weather, including staying hydrated, avoiding strenuous activity during hot hours of the day, and seeking cool environments when necessary [10,11]. On the other hand, physical activity (PA) has long been studied as a protective factor against falls, with numerous studies highlighting its role in improving balance, strength, and coordination, all of which contribute to a reduced risk of falling in older adults and individuals with various health conditions. Engaging in regular PA, such as balance exercises and aerobic activities, can enhance physical fitness and functionality, hence significantly lowering the incidence of falls [12–14].

### 1.2. Related Work

Limited research has been conducted on the relationship between extreme heat, PA, and falls among LOAs despite growing concerns. LOAs with limited mobility are less likely to engage in PA, which is a key factor in maintaining overall health, healthy aging, and preventing chronic conditions [15]. A recent study revealed that extreme heat negatively affects PA and sleep pattern [16]. In other words, rising temperature has been found to lead to an increase in sedentary behavior (SB). This finding is important given the emerging evidence that SB is a risk factor for multiple disorders, including cognitive decline, weight gain and obesity, and cancer risk [16]. These negative effects are especially concerning for individuals with limited access to air conditioning, particularly in lower-income neighborhoods. Furthermore, individuals with cognitive impairments such as Alzheimer’s disease, dementia, or hereditary and degenerative diseases of the central nervous system were associated with elevated risk of mortality on extremely hot days and increases in warm-month temperatures [17,18]. Numerous studies consistently show that LOAs or older adults who reside in rural areas are significantly less likely to participate in regular PA compared to their higher-income peers, mainly due to barriers such as limited access to facilities, transportation difficulties, and a lack of awareness about the benefits of exercise [19–21]. Environmental factors further enhance these challenges, as high temperature influences PA levels, limits opportunities for physical movement, and increases health risks such as falls [11,22]. Nonetheless, many LOAs have limited control over their built environments, restricted access to healthcare, and fewer resources to advocate for community-level changes that could help mitigate the impact of elevated temperature on their health and well-being. This disparity in maintaining an active lifestyle further exacerbates health inequities within the older population.

Traditional statistical approaches, such as mixed-effect and time-series modelings, have been widely used to examine the impact of ambient temperature and PA [16,22–24]. For example, Tang et al. proposed a Zero-inflated Smoothing Spline method to model single-cell temporal data, which encompassed two components for modeling gene expression patterns over time and handling excessive zeros [25]. However, these regression methods often struggle to capture the sophisticated relationships and interactions that may exist among variables. In contrast, machine learning techniques offer greater flexibility in modeling intricate patterns and dependencies without imposing strict parametric assumptions. Many studies utilized the supervised models similar to traditional regression models, including support vector machine, tree-based and boosting algorithms, to electronic health record data to predict fall events in the older population [26–28]. Other studies employed unsupervised models; for instance, Yuwono et al. utilized a single waist-worn tri-axial accelerometer, combining digital signal processing, clustering, and neural network classifiers—specifically, an Augmented Radial Basis Function neural network with a Multilayer Perceptron—to classify fall and activity signals, demonstrating improved sensitivity and specificity [29]. Specifically, recent advancements in graphical models include the work by Khan et al. [30], who introduced picture fuzzy directed hypergraphs to enhance decision-making in complex environments such as hazardous chemical management, and the study by Li et al. [31], which proposed a novel method for estimating multi-attribute Gaussian copula graphical models, extending the application of graphical models to more flexible and heterogeneous data structures. Nevertheless, few studies have successfully combined statistical and machine learning approaches, making it difficult to fully uncover more complex, nonlinear associations and gain a deeper understanding of the dynamics influencing fall in the context of temperature variations. Hence, integrating a hybrid framework allows researchers to validate findings through interpretable parametric models while leveraging the power and flexibility of machine learning to detect subtle patterns, interactions, and potential high-order effects, which presents a promising avenue for advancing fall risk research.

### 1.3. Observation and Hypothesis

To better understand how outdoor temperature affects older adults in real life, we conducted a pilot study exploring their daily habits, challenges, and fall risk during hot weather. In this pilot study (N=41) [32], LOAs are more likely to have less time spent on intense physical activity but a higher number of falls in summer months compared to fall (onsite survey interviews with a focus group of 21 individuals; most were Spanish speakers). About 71% felt uncomfortable on a hot day, 22% reduced PA, 22% did not use air conditioning and only 36% stayed hydrated. The top three barriers were health issues (20%), lack of time (11%), and transportation difficulty (9%). More than 50% of participants stated that heat or hot weather affected their sleep pattern, which aligns with a previous study [16], in addition to daily food intake and hydration; 32% reported that heat impacted their social connections, and 47% had no access to community resources [32].

**Figure 1.**
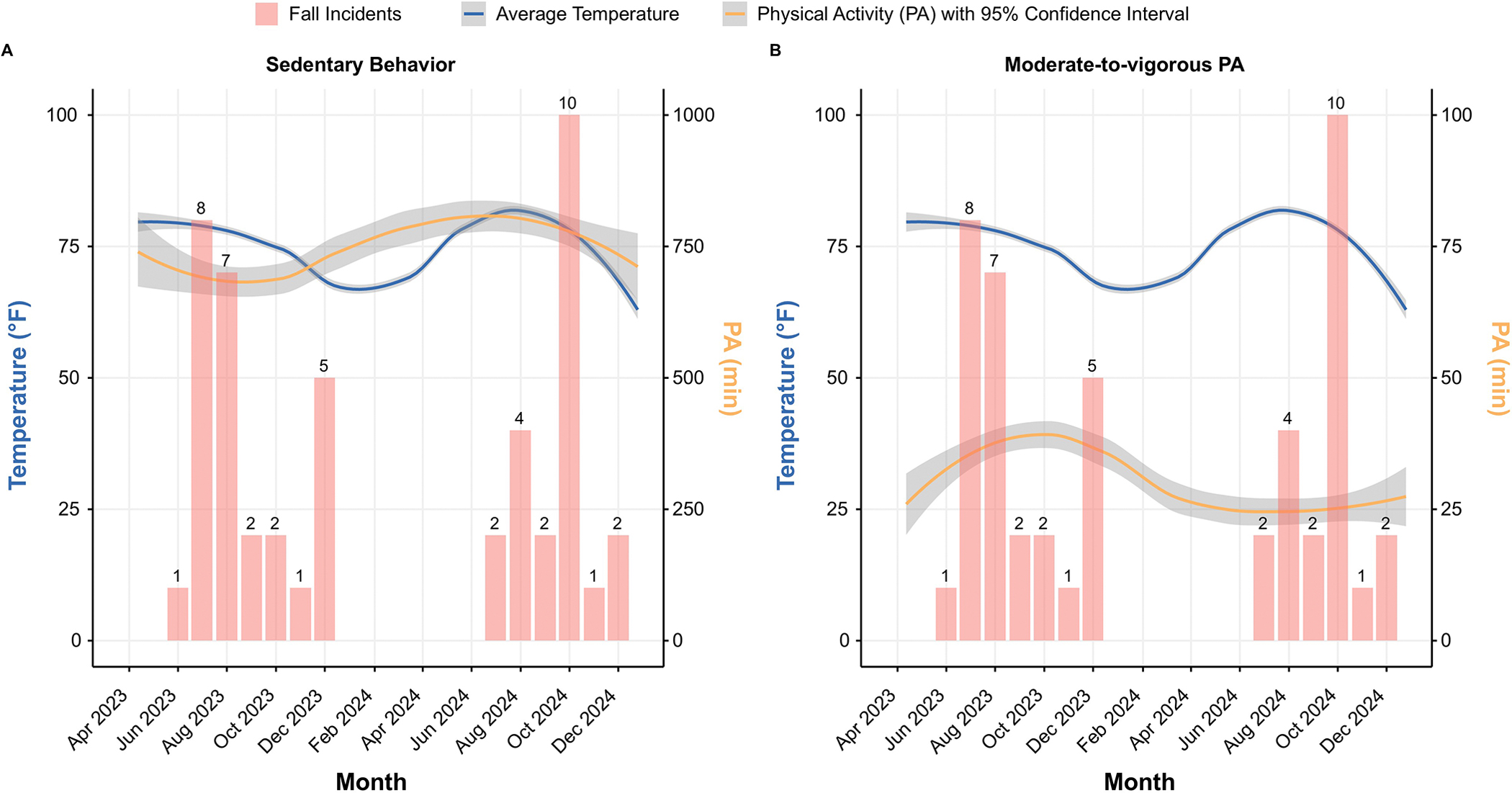
Observed time spent in physical activity (PA) (minutes), temperature (Fahrenheit), and fall incidents during the study period (April 2023–December 2024). (**A**) Sedentary behavior. (**B**) Moderate-to-vigorous PA.

Building on these results, we expanded the analysis to a larger dataset (N=304) to examine seasonal trends in PA, temperature, and fall incidents over time. As illustrated in Figure 1, the fall counts, represented by red bars, show notable peaks in October 2024 (ten incidents), July 2023 (eight incidents), and August 2023 (seven incidents). Between April 2023 and December 2024, the average daily temperature (blue line) follows a sinusoidal pattern, reaching its highest in the summer months (approximately 80 °F) and its lowest levels during winter (around 60 °F). Meanwhile, the PA levels represented by the orange line with a 95% confidence interval demonstrates a fluctuating relationship with temperature. Particularly, time spent on moderate-to-vigorous physical activity (MVPA) was increasing during transitional months (August–October 2023), and decreasing during summer time (Figure 1B). On the contrary, time spent on SB peaked during the summer months of 2024 and maintains an inverse relationship with temperature, rising during colder months and decreasing in warmer periods (Figure 1A). Although fall events tend to be more frequent during transitional seasons, such as August–October 2023 and October–December 2024, they do not always coincide with peak PA levels, suggesting that other factors may contribute to fall risks.

### 1.4. Contributions

**Figure 2.**
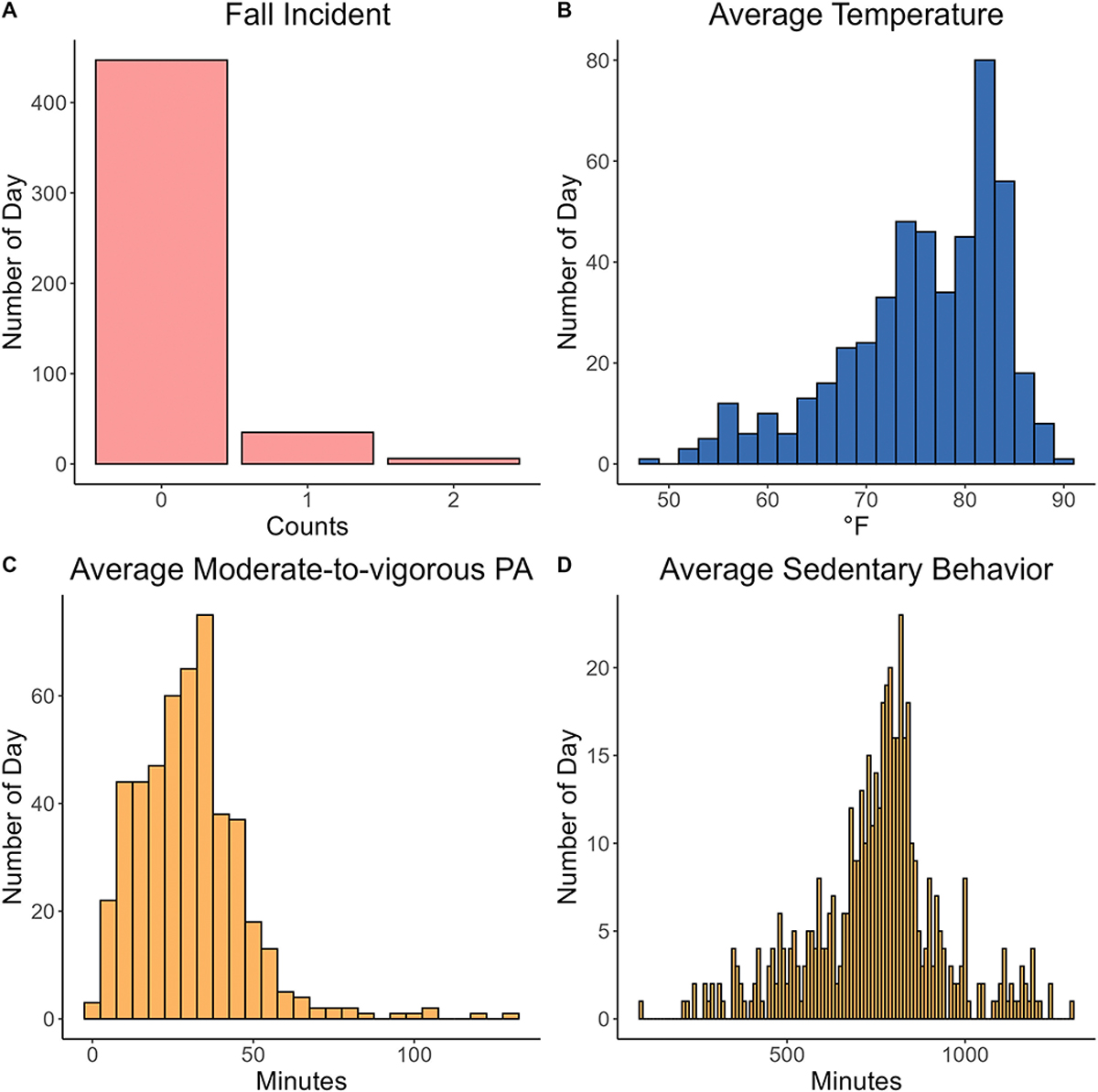
Histograms of (**A**) fall incident, (**B**) average moderate-to-vigorous PA time (minute), (**C**) average sedentary behavior time (minute), and (**D**) average outdoor temperature (°F). Colors were chosen for visual clarity and are used consistently throughout: pink = fall incident, dark blue = temperature, and orange = PA (MVPA, SB).

This study aims to examine the role of ambient outdoor temperature in physical activity engagement and fall risks with a longitudinal cohort of older adults. Based on our observation, we hypothesize that higher temperatures will be associated with lower PA level or higher sedentary times, which in turn leads to increased risk of falling or fall events among LOAs. Furthermore, we explore sociodemographic factors (age, gender, race/ethnicity, education level, living condition, financial difficulty, and self-rated health) in these associations. However, fall incidents were infrequent and highly sparse in our data, as illustrated in Figure 2. The sparsity in event occurrence, along with the excess zeros in the dataset, may lead to overdispersion, where the variance exceeds the mean.

We proposed a zero-inflated Poisson (ZIP) regression model, which is well suited for count data with an overabundance of zero values, to address the aforementioned issues. ZIP regression is commonly used in public health research due to its ability to account for these structural zeros, assuming that with probability π the only possible observation is 0, while with probability 1-π, the count follows a *Poisson*(*λ*) distribution [33,34]. Although the ZIP regression reflects linear relationships, a more comprehensive and rigorous investigation is needed to assess the impacts of extreme heat on falls as there is recent evidence of the mixed and multi-factorial nature of fall events, both at individual and community levels [35].

To capture complex, potentially nonlinear relationships within the data, we developed and employed a mixed undirected graphical model (MUGM), an advanced machine learning analytical approach tailored for this purpose. MUGM, an unsupervised machine learning technique, is increasingly applied in healthcare and public health research due to its flexibility and interpretability [36,37]. The application of MUGM in this study offers several key technical contributions. Unlike traditional regression methods, MUGM offers an innovative framework for addressing the challenges in high-dimensional statistics, where the number of features is significantly larger than the number of observations, and for uncovering complex, nonlinear or high-order associations between variables, without relying on strict parametric assumptions such as the distribution type, the parameter additivity, the predictor linearity, or homoscedasticity [38]. MUGM also enables the integration of mixed data types (continuous and categorical), making them ideal for analyzing diverse health-related variables, especially in older adult populations. Moreover, graphical models can visually map out interactions between variables and enhance interpretability, providing valuable insights into the multifaceted determinants of falls.

This two-pronged analytical approach demonstrates the complementary effectiveness of both traditional statistical and advanced machine learning methods, providing a more nuanced understanding of the relationships between factors associated with fall incidents. As we aim to explore adaptation mechanisms in the context of socio-environmental disparities, the hybrid approach strengthens our ability to address fall prevention holistically, bridging the gap between theoretical insights and practical applications.

The remainder of this paper is organized as follows: Section 2 introduces the study design, participants, and the proposed methods for fall incident modeling and prediction. The main analysis and results are described in Section 3. Lastly, we discuss the principal findings, limitations of the study, and conclude the article with future work in Section 4.

## 2. Materials and Methods

We introduce the study design, participant recruitment, and study variables, followed by data analysis using ZIP regression to model and estimate fall incidents in LOAs. Lastly, we apply MUGM to systematically identify possible (nonlinear) relationships among daily PA, temperature, and fall counts in older adults.

### 2.1. Study Design and Participants

A total of 304 community-dwelling individuals aged 60 and older, from low-income communities and living in free-living conditions, were recruited in the central region of Florida, USA. The study ranged from April 2023 to December 2024 with 11 independent living communities and senior centers. The inclusion criteria consist of (1) meeting the low-income criteria based on the 2019 Poverty Guidelines [39], (2) having the ability to walk with or without an assistive device but independently from the assistance of another person, (3) having no cognitive impairment, (4) living independently in homes or apartments, and (5) being fluent in English and/or Spanish. Participants completed qualitative interviews, including but not limited to self-report questionnaires of sociodemographic and self-rated health. In addition, each individual was provided a wrist-worn wearable device, named ActiGraph GT9X Link (ActiGraph Corp, Pensacola, FL, USA) and given instructions on how to wear it for 7 consecutive days.

### 2.2. Study Variables

Sociodemographic information was obtained through self-report questionnaires, as described in [40]. Participants provided their age in years and their gender as a binary categorical variable. Race and ethnicity consisted of non-Hispanic Asian, non-Hispanic African American, Hispanic, and non-Hispanic White. The education level was categorized into two levels: high school or below, and college or higher. Although the financial difficulty was at multiple levels, it was grouped and simplified into adequate or less, and more than adequate. Participants were classified as either living alone or living with others. Self-rated health status was measured using a five-point Likert scale, and individuals were grouped into excellent or very good (class 1 and 2), and good or below (remaining classes).

Participants wore the accelerometer on the non-dominant wrist for 7 consecutive days, which continuously monitored various physiological metrics, including PA. The devices were set up to record accelerations at 30 Hz. The raw accelerometer data were processed using the R package GGIR (version 2.4.0) [41], which provided daily estimates of time spent in different activity intensities: sedentary behavior (SB), light-intensity physical activity (LPA), and moderate-to-vigorous physical activity (MVPA). Particularly, the Euclidean Norm Minus One threshold was used to classify the time spent in SB (<30 milligravity (mg)), LPA (30–99 mg), and MVPA (≥100 mg) [42,43].

Daily temperature data for Central Florida (Orlando, FL, USA), spanning the study period (April 2023 to December 2024), were retrieved from the publicly available Visual Crossing weather data repository [44]. The recorded average daily temperatures ranged from 47.5 to 89.4 °F and were incorporated into subsequent analyses.

### 2.3. Data Analysis

Descriptive statistics of participant characteristics are presented as mean and standard deviation for continuous variables, and as frequency and percentage for categorical variables. Missingness was observed and imputed using the Kalman smoothing method for time-series data [45]. All analyses were performed using the R Statistical Software (version 4.4.1) [46] and a *p*-value < 0.05 was considered statistically significant.

#### 2.3.1. Zero-Inflated Poisson (ZIP) Regression

The most widely used regression model for count data, particularly to model fall events, is probably the Poisson regression. For observation i=1,⋯,n, let $Y={\left(Y_{1},\cdots ,Y_{n}\right)}^{⊤}$ denote the vector of independent response variables, and let $X\in R^{n\times p}$ denote the matrix with rows $x_{i}=\left(x_{i1},\cdots ,x_{ip}\right)$. Then,

(1) $$Y_{i}∼Poisson\left(\lambda_{i}\right),\;where\;\lambda_{i}=exp\left(\beta_{0}+\beta_{1}x_{i1}+\cdots +\beta_{p}x_{ip}\right).$$

The above generalized linear model incorporates a Poisson random component, a linear predictor function, and a log link [34]. Poisson regression models are commonly considered for count responses and assume that the count follows Poisson distribution, with mean λ equaling its variance. The estimated coefficients indicate the expected change in the log of the mean count for each unit increase in the corresponding predictor. Instead of using the log-transformed mean count directly, the inverse of Equation (1) is commonly applied by exponentiating the model coefficients to derive rate ratios [47]. To account for the excess amount of zeros, an extension model, known as the zero-inflated Poisson (ZIP), model is introduced. This model includes two sets of regression parameters: one for estimating the Poisson mean and another for determining the probability of an excess zero, providing a latent class interpretation [33]. Thus, we assume that

(2) $$Y_{i}=\left\{\begin{array}{ll} 0 & with\;probability\;\pi_{i}+\left(1-\pi_{i}\right)e^{-\lambda_{i}}\\ k & with\;probability\;\left(1-\pi_{i}\right)e^{-\lambda_{i}}\frac{\lambda_{i}^{k}}{k!},\;k=1,\;2,\;\ldots \end{array}\right.$$

where π is the probability of being a zero. The parameters $\lambda_{i}$ and $\pi_{i}$ satisfy $logit\;\left(\lambda_{i}\right)=x_{i}^{⊤}\beta$ and $logit\left(\pi_{i}\right)=z_{i}^{⊤}\eta$, in which $\beta ={\left(\beta_{1},\cdots ,\beta_{m_{1}}\right)}^{⊤}$ is the $\left(m_{1}\times 1\right)$ vector of parameters associated with the Poisson distribution; $\eta ={\left(\eta_{1},\cdots ,\eta_{m_{2}}\right)}^{⊤}$ is a $\left(m_{2}\times 1\right)$ vector of parameters associated with the excess zeros; and $x_{i\left(1\times m_{1}\right)}^{⊤}$ and $z_{i\left(1\times m_{2}\right)}^{⊤}$ are the vectors of covariates for the *i*th individual for Poisson and excess zeros, respectively [33,48]. Hence, the model assumes that with probability $\pi_{i}$, the only possible observation is 0, and with probability $1-\pi_{i}$, the count follows a ${Poisson(\lambda }_{i})$ distribution. In this study, all variables, including average outdoor temperature, physical activity intensities (MVPA, SB), and sociodemographic factors, were included in the Poisson count component of the ZIP model, while no predictors (intercept-only) were used in the zero-inflation component due to the limited number of fall incidents. The Poisson count component estimates how these variables influence the expected number of falls among participants who are at risk. In contrast, the zero-inflation component models the baseline probability that a participant belongs to the structural zero group, that is, individuals with no underlying risk of falling during the study period.

The likelihood function for this zero-inflated model is then derived as

(3) $$ℒ\left(\lambda ,\pi |Y\right)=\prod_{Y_{i}=0}\left((\frac{\pi_{i}}{1\;-\;\pi_{i}}+e^{-\lambda_{i}})\left(1-\pi_{i}\right)\right)\prod_{Y_{i}>0}\left(\left(1-\pi_{i}\right)e^{-\lambda_{i}}\frac{\lambda_{i}^{Y_{i}}}{Y_{i}!}\right).$$

In our study, no participants experienced any falls at the start of the observation period, meaning that the count of fall incidents for all subjects begins at zero. However, we hypothesize that various covariates, such as PA, temperature, and sociodemographic factors, may influence the likelihood of falls, potentially increasing the risk of incidents. The outcome of interest, fall count, is assumed to follow a Poisson distribution, and the initial full model is specified as

(4) $${Fall}_{i}∼\mathrm{ZIP}\left(\lambda_{i},\pi_{i}\right)$$

where $\lambda_{i}=exp\left(x_{i}^{⊤}\beta \right)$ is the expected count of falls for participant i from the Poisson component, and $\pi_{i}=\frac{exp(\eta )}{1+exp(\eta )}$ is the probability that participant i belongs to the structural zero group, modeled with an intercept-only zero-inflation component. Here, $x_{i}$ includes all covariates listed above, and β and η are the corresponding coefficients in the Poisson and zero-inflation components, respectively.

We report the relative risk (RR) estimates derived from the count component to interpret associations between covariates and fall likelihood. All covariates were standardized prior to analysis. Model selection was performed on the count component using backward elimination based on the Akaike Information Criterion (AIC) and Bayesian Information Criterion (BIC), systematically removing non-significant predictors to achieve a more parsimonious model. The ZIP model was implemented in R using the pscl package [49] and the reproducing code is provided in the Supplementary Materials. The ZIP model is summarized in Algorithm 1.

**Algorithm 1:** Pseudocode for Zero-Inflated Poisson (ZIP) Regression

**Input:** Data matrix X, response vector Y
**Output:** Parameter estimates $\overset{ˆ}{\beta }$ and $\overset{ˆ}{\eta }$
1: Estimate the probability that each zero in Y comes from the zero-inflation component using Equation (2).
2: Estimate the parameters $\beta^{\left(t\right)}$ and $\eta^{\left(t\right)}$ by maximizing the likelihood using Equation (3).

#### 2.3.2. Mixed Undirected Graphical Model (MUGM)

The MUGM was utilized to examine the associations between fall-related risk factors in a dataset consisting of both continuous and categorical variables. This graphical-based approach leverages machine learning to provide flexible and adaptable solutions for handling diverse data types [38,40]. A graphical model is an unsupervised machine learning technique that can uncover the joint probability distribution as well as the strength and direction of relationships among a set of random variables [50]. In undirected graphical models, also referred to as Markov random fields or Markov networks, each node corresponds to a random variable, and the undirected edges between nodes represent associations between those variables. If two nodes are connected by an edge, they are considered adjacent, denoted as X∼Y. Conversely, if there is no edge between two nodes, it indicates that the variables are conditionally independent, given all other variables, and represented as X⊥Y|rest.

The probability density function f of an undirected graph is derived as

(5) $$f\left(x\right)=\frac{1}{U}\prod_{c\in 𝒞}\psi_{c}\left(x_{c}\right),$$

where 𝒞 indicates a collection of cliques in the graph, $\psi_{c}\left(x_{c}\right)$ is a non-negative potential function of the input nodes, and U is a normalization constant obtained by integrating or summing the product with respect to $x_{c}$. A graphical model can specify higher-order dependencies within a joint probability distribution [50]; however, only pairwise interactions are presented in this study. The model includes the following variables:

- Continuous variables: Daily outdoor temperature, MVPA, SB, age, and fall incidents.
- Categorical variables: Gender, race/ethnicity, education level, living condition, financial difficulty, and self-rated health.

The mixed undirected graphical model (MUGM) can learn from a mix of continuous and discrete variables [38,50,51]. The density of pairwise mixed graphical model for p continuous variables x and q categorical variables z is formulated as

(6) $$f(x,z;\Psi )\propto exp\left(\sum_{s=1}^{p}\sum_{t=1}^{p}-\frac{1}{2}\beta_{st}x_{s}x_{t}+\sum_{s=1}^{p}\alpha_{s}x_{s}+\sum_{s=1}^{p}\sum_{j=1}^{q}\rho_{sj}\left(z_{j}\right)x_{s}+\sum_{j=1}^{q}\sum_{r=1}^{q}\phi_{rj}\left(z_{r},z_{j}\right)\right)$$

where $x_{s}$ and $x_{t}$ denote the *s*th and *t*th of p continuous variables, respectively; and $z_{r}$ and $z_{j}$ denote the *r*th and *j*th of q discrete variables, respectively. The joint model parameter space is $\Psi =\left[\left\{\beta_{st}\right\},\left\{\alpha_{s}\right\},\left\{\rho_{sj}\right\},\left\{\phi_{rj}\right\}\right]$. The model parameters include $\beta_{st}$, which represents continuous–continuous edge potential;$\;\alpha_{s}$, which signifies continuous edge potential; $\rho_{sj}\left(z_{j}\right)$, which denotes continuous–discrete edge potential; and $\phi_{rj}\left(z_{r},z_{j}\right)$, which indicates discrete–discrete edge potential. The MUGM extends two well-established single-modal models: when all variables are continuous, it simplifies to a multivariate Gaussian distribution and to Ising models when all variables are discrete [51,52].

The parameters in Ψ can be estimated by minimizing the negative log-likelihood:

(7) $$ℒ(\Psi )=-\sum_{i=1}^{n}log\;f\left(x_{i},z_{i};\Psi \right)$$

where

(8) $$log\;f\left(x_{i},z_{i};\Psi \right)=\sum_{s=1}^{p}\sum_{t=1}^{p}-\frac{1}{2}\beta_{st}x_{s}x_{t}+\sum_{s=1}^{p}\alpha_{s}x_{s}+\sum_{s=1}^{p}\sum_{j=1}^{q}\rho_{sj}\left(z_{j}\right)x_{s}+\sum_{j=1}^{q}\sum_{r=1}^{q}\phi_{rj}\left(z_{r},z_{j}\right)-\log U\left(\Psi \right).$$

Here, U(Ψ) is the normalizing constant that ensures the joint density $f\left(x_{i},z_{i};\Psi \right)$ integrates to one over the support.

The edges in MUGM are selected using penalization to ensure a sparse graphical model. The likelihood is maximized subject to edge penalization by solving the following regularized optimization problem:

(9) $$\underset{\Psi }{min}\;{ℒ}_{\gamma }(\Psi )=ℒ(\Psi )+\gamma \left(\sum_{t<s}\left|\beta_{st}\right|+\sum_{s,j}{\left\|\rho_{sj}\right\|}_{2}+\sum_{r<j}{\left\|\phi_{rj}\right\|}_{F}\right)$$

where the tuning parameter γ controls the regularization. Since there are three types of edges in pairwise mixed model ($\beta_{st},\rho_{sj}$ and $\phi_{rj}$), we use $\ell_{1}$-norm for scalars, $\ell_{2}$-norm for vectors and Frobenius norm for matrices. An accelerated proximal gradient method can then be implemented to solve this optimization problem [51].

The Extended Bayesian Information Criterion (EBIC) was employed for model selection as it outperforms the standard BIC in high-dimensional feature spaces [53]. Using EBIC as the model performance metric, 10-fold cross-validation was employed to select the optimal regularization parameter $\gamma_{opt}$. Cross-validation method involves randomly splitting the data into ten subsets, estimating a graph from nine of them while testing the negative log-likelihood on the remaining subset. This procedure is repeated with each set acting as the testing fold, resulting in ten performance metrics, and the optimal regularization parameters were selected based on the best performance. The entire dataset is then used to retrain the model for final reporting. Other methods for selecting the regularization parameter consist of AIC, BIC, Stability Approach to Regularization Selection [54], and Stable Edge-specific Penalty Selection [52]. The resulting conditional dependency graph allowed us to visualize the structure of fall-related risks, for instance, whether MVPA is more connected to temperature or to sociodemographic features like gender or age.

The MUGM was performed using the mgm package (version 1.2–14) [55]. The resulting graph was visualized with the qgraph package (version 1.9.5) [56] and the reproducing code is provided in the Supplementary Materials. The edges in the graph, which represent associations between two variables while controlling for all other variables, are weighted, and their strength is indicated by regression coefficients. The thickness of these edges corresponds to the strength of these associations. Furthermore, the edge colors signify the direction of the relationships: green denotes positive associations and red denotes negative associations. The weighted adjacency matrix, which contains the regression weights for each pair of variables, was used to construct the graph, allowing for a more detailed representation of the interdependencies between variables. The MUGM method is summarized in Algorithm 2.

**Algorithm 2:** Pseudocode for Mixed Undirected Graphical Model (MUGM)

**Input:** A dataset features X, including both continuous and categorical variables
**Output:** Estimated undirected graph
1: Train the mixed undirected graphical model using Equation (9).
2: Perform 10-fold cross-validation to select the optimal regularization parameter $\gamma_{\text{opt}}$ based on EBIC.
3: Update the graphical model using the selected optimal $\gamma_{\text{opt}}$.
4: Visualize the undirected graph with nodes of the same category positioned closely.

## 3. Results

The mean age of participants is 74.71 years (SD = 7.23), with a majority female population (86.2%). Among them, 38.5% are Non-Hispanic African American, 29.6% are Hispanic, 22.7% are Non-Hispanic White, and 7.2% are Non-Hispanic Asian. Regarding education level, 53.6% attended high school or below, while 45.8% had some college or higher. Most participants (77.3%) report more than adequate financial resources, and 55.3% live alone. Moreover, 63.8% of the participants describe their general health as good or below. Accelerometer data reveal that participants engage in an average of 729.84 min a day of SB, 204.70 min a day of light-intensity physical activity (LPA), and 31.35 min a day of MVPA. Notably, 86.8% of participants do not experience any fall throughout the study period, although 39 of them have 1 or 2 fall events (10.9% and 2%, respectively). Table 1 presents the descriptive characteristics of the 304 older adults. A correlation matrix between continuous variables are provided in Table 2. Specifically, temperature is significantly positively correlated with fall (*ρ* = 0.13, *p* < 0.001), suggesting that higher ambient temperatures may be associated with increased fall risk. SB is significantly negatively correlated with MVPA (*ρ* = −0.10, *p* < 0.001) and positively with age (*ρ* = 0.09, *p* < 0.001), indicating that older individuals tended to be more sedentary and less physically active. MVPA shows a moderate negative correlation with age (*ρ* = −0.304, *p* < 0.001), consistent with age-related declines in activity levels. Additionally, MVPA is inversely associated with fall incident (*ρ* = −0.036, *p* = 0.002).

Table 3 presents the results of the ZIP regression examining the associations between average temperature, mean MVPA and SB times, and sociodemographic factors with fall counts. Exponentiating the coefficient for mean MVPA yields the estimate of fall counts at *e*^−0.105^ = 0.9, indicating that a higher mean MVPA is significantly associated with fewer fall incidents (95% CI: (0.816, 0.993), *p* = 0.037) after adjusting for other variables. This suggests that increased engagement in MVPA may serve as a protective factor against falls. On the other hand, higher temperatures are strongly associated with increased fall incidents (*e*^0.550^ = 1.733, 95% CI: (1.581, 1.901), *p* < 0.0001), potentially indicating seasonal effects, where environmental factors such as elevated temperatures as well as changes in activity patterns or behaviors contribute to fall risk. Moreover, being Non-Hispanic White is significantly associated with higher fall events (*p* < 0.0001), which may reflect racial or ethnic differences in unmeasured health conditions, activity patterns or access to healthcare. The significant negative interaction between temperature and SB (*p* < 0.0001) suggests that although higher temperatures increase fall risk, this effect is mitigated among older adults with increased sedentary behavior. Similarly, the negative interaction between two PA levels (*p* = 0.0004) may imply that engaging in both activity types may have a compensatory or protective effect against falls, though further research is needed to validate this relationship. In addition, the model revealed a statistically significant zero-inflation component, with an intercept estimate of −0.682 (*p* = 0.017) (Supplementary Materials). This indicates that approximately 33.6% of the zeros in the outcome variable are likely structural zeros, that is, not generated by the Poisson count process. Since no covariate was included in the zero-inflation part, the probability of being an excess zero is assumed to be constant across observations. This result supports the appropriateness of using a zero-inflated model over a standard Poisson model for these data.

**Figure 3.**
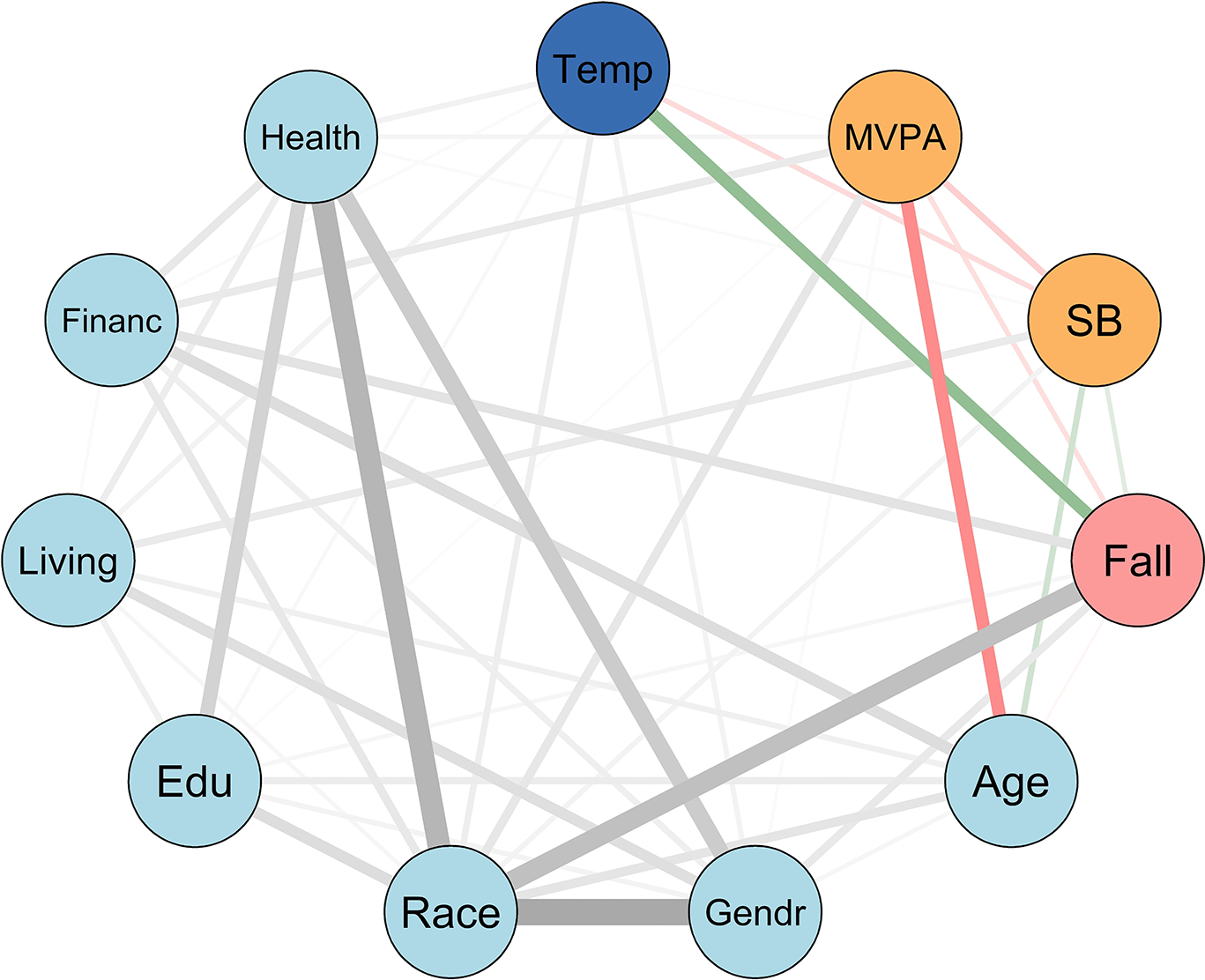
Mixed undirected graphical model estimated the associations among fall incident (Fall), average temperature (Temp), physical activity intensities (MVPA and SB), and sociodemographic factors (Age, Gendr, Race, Edu, Living, Financ, and Health) of 304 low-income older adults. Edges represent conditional dependencies and their thickness reflects the strength of the association: thicker edges indicate stronger associations. Edge color indicates direction: green for positive and red for negative. Gray edges represent relationships involving at least one categorical variable, whose sign cannot be determined. Node colors were chosen for visual clarity and are used consistently throughout to represent different variable categories: dark blue = temperature, orange = PA (MVPA, SB), pink = fall events, and light blue = sociodemographic factors.

Given the significant effects of PA and average temperature on fall counts observed in the ZIP regression, we extended our analysis using the MUGM to capture more complicated, potentially nonlinear associations. The MUGM included 11 variables (nodes), including fall events, and the nodes are color-coded by category: the fall events variable is shown in pink, PA measures (MVPA and SB) in orange, temperature in dark blue, and sociodemographic variables in light blue. In general, 79.3% of all possible edges were non-zero, suggesting a dense and interconnected network. Each variable was connected to at least one other through a non-zero pairwise association, and a total of 46 edges were identified (Figure 3). Of these, 3 edges (6.5%) demonstrated moderate associations (defined as edge weights between 0.4 and 0.7), while the remaining 43 edges (93.5%) showed weak associations (edge weights less than 0.4). We considered any edge with a weight below 0.01 as negligible and excluded these from the final visualization. Full details of the weighted pairwise associations can be found in Table 4. As indicated, race/ethnicity, gender, and fall incidents are among the strongest weights in this network.

Several notable patterns emerge, including a positive association between fall events and temperature, suggesting that higher temperatures may be linked to increased fall risk, and a moderate association between fall events and race, further supporting findings from our ZIP regression. Falls were also negatively associated with age, implying that older individuals reported fewer falls, which may reflect more cautious behavior, less mobility, or under-reporting. Additionally, falls showed a moderate association with race/ethnicity, as indicated by a thick gray edge, pointing to potential disparities linked to racial or socio-environmental factors. Age was moderately and negatively associated with MVPA, indicating a decline in PA with advancing age. MVPA is weakly negatively linked to falls, while SB is weakly positively associated with falls. These trends suggest that both higher PA and lower sedentary time may be modestly protective against falls. Sociodemographic variables form a dense cluster, with race/ethnicity serving as a central node connected strongly to self-rated health, gender, and education level. Self-rated health is also related to education level and gender, highlighting the interconnectedness of social determinants. Meanwhile, participant age was moderately associated with financial difficulty.

For each node, the optimal regularization parameter γ and corresponding EBIC values used for model selection are reported in Table 5. Lower EBIC values indicate that a variable is more stably and informatively connected within the graphical structure. For example, variables such as gender (244.44), general health (402.26), and living status (415.42) had comparatively low EBIC values, implying simpler and more stable conditional associations with other variables. In contrast, race had the highest EBIC value (1197.07), which may reflect its multicategorical structure and a more complex conditional model with multiple parameters. Despite its high EBIC, race may still exhibit strong associations with other variables, but its prediction from the rest of the network is more complex. These EBIC values guided the selection of the regularization parameter *γ* and helped refine the final network structure.

## 4. Discussion

### 4.1. Principal Results

The findings of this study reveal critical associations between PA, temperature, and fall incidents among older adults, highlighting the multi-factorial nature of fall risk. Notably, higher PA levels were significantly associated with fewer fall incidents, suggesting that increased engagement in PA, especially MVPA, may serve as a protective mechanism against falls. This aligns with prior research emphasizing the benefits of PA in maintaining balance, strength, and overall mobility in older adults [12,15,57].

In contrast, elevated temperatures were strongly linked to a greater risk of falls, which could reflect seasonal influences where environmental conditions, changes in activity patterns, or physiological responses contribute to increased susceptibility [16,17,58,59]. This seasonal effect may be influenced by factors such as dehydration, heat-induced fatigue, or increased outdoor activity during warmer months, all of which could contribute to instability and higher fall risk. Moreover, biological mechanisms in older adults such as impaired thermoregulation due to declining sweat gland function and reduced circulation efficiency [9], as well as heat-related cognitive decline [18], may further exacerbate the likelihood of fall. Additionally, many older individuals have chronic medical conditions or take prescription medicines that change normal body responses to heat, further affecting the body’s ability to control its temperature or sweat. These physiological impairments and preexisting conditions can reduce balance, coordination, and physical performance, making older adults particularly vulnerable and more likely to experience falls during hotter periods. However, this effect is mitigated among individuals with more sedentary time. Similarly, interactions between different physical activity levels imply a potential compensatory effect in fall prevention, which require further exploration into how various movement patterns collectively influence fall susceptibility.

Further findings were obtained from the MUGM, which effectively uncovered associations among interrelated factors, reinforcing the complicated nature of fall risk. MUGM, which is not only limited to linear effects, revealed both linear and nonlinear relationships, showing that every variable in the network exhibited at least one pairwise association. Falls were strongly correlated with race and positively associated with ambient temperature, aligning with the trends previously identified through the ZIP regression model. Notably, age demonstrated multiple associations, including a negative correlation with MVPA and moderate relationships with race and financial status, suggesting that both biological and socioeconomic factors contribute to fall vulnerability. Additionally, general health perception was strongly connected to race, gender, and education level, as supported by previous research [60,61], emphasizing the broader sociodemographic influences on overall health outcomes. The strength of the MUGM approach lies in its ability to offer a comprehensive view of how behavioral, environmental, and demographic variables interact, both directly and indirectly, to shape fall risk, making it a powerful tool in complex data.

Given these findings, it is essential to develop practical strategies for reducing fall risk in hot environments while maintaining PA levels, particularly in resource-limited settings or communities facing health disparities. A key approach is to balance PA with thermal safety. Encouraging older adults to engage in PA during cooler times of the day, such as early mornings or evenings, can help mitigate heat-related fatigue and dehydration risks [10,62]. Adequate hydration (i.e., before, during, and after activity) is also crucial in preventing heat stress and maintaining both cognitive and physical function. Furthermore, modifying PA routines to include indoor or shaded activities, such as chair exercises or walking in climate-controlled spaces (e.g., malls, community centers), can help sustain engagement while minimizing fall risks [12]. Simple, cost-effective interventions, such as proper footwear, balance training, and environmental modifications (e.g., reducing trip hazards, using cooling fans), can also further enhance stability and safety in warm conditions [11,63]. More importantly, community-based programs that integrate PA with fall prevention education, and social determinants of health into fall risk assessment, especially in underserved areas, can empower older adults with practical strategies to stay active and safe despite environmental challenges.

### 4.2. Limitations and Future Work

Despite this study offering valuable insights into the associations between different factors and fall incidents, it is important to acknowledge certain limitations. First, reliance on self-reported fall incidents may be prone to recall bias, as participants may under-report or misremember fall events, leading to potential misclassification. Similarly, sociodemographic variables, such as financial status, was self-reported, which could be subject to response biases. Objective measures of falls, such as wearable sensors or medical records, could enhance the accuracy of fall assessment in future research. Second, although temperature was found to be a significant predictor of falls, other important factors such as humidity, precipitation, heat index, or weather-related factors such as hydration status, heat-related fatigue, psychological changes, or indoor versus outdoor activity participation, were not considered in the analysis. Similarly, unmeasured confounders such as underlying health conditions (e.g., diabetes, cardiovascular disease), medication use (e.g., those affecting thermoregulation or cognition), or social determinants of health (e.g., housing quality, access to resources) were not included in this study. These factors may independently or interactively play a crucial role in fall risk; thus, their omission may introduce bias into the observed associations. Failing to control for such confounders increases the risk of overestimating or underestimating the true effect of temperature on fall risk. Future studies should carefully consider a broader range of environmental, behavioral, and individual-level variables to improve the validity and robustness of findings.

Additionally, the analytical methods used do not fully leverage longitudinal techniques that account for temporal dependencies and within-subject variations over time. This study primarily relies on statistical associations that reflect cross-sectional relationships at given time points rather than capturing dynamic changes in fall risk factors over time. Consequently, the findings may not adequately reflect the causal relationships or the long-term trajectories of PA, environmental exposures, and fall incidents in LOAs. Without modeling time-varying effects, it remains unclear whether certain factors directly contribute to falls or whether underlying confounding variables influence these associations. Further research utilizing more sophisticated longitudinal models, such as mixed-effects modeling, time-series analyses, or causal inference frameworks that incorporate temporal structure (e.g., marginal structural models), is needed to assess temporal relationships between individual behaviors and environmental exposures.

Another limitation lies in the complexity of the MUGM analysis. The inclusion of categorical variables required certain methodological assumptions, which may have affected the results. Additionally, the observed strengths of the associations within the graph were generally weak to moderate, suggesting that unmeasured factors or key variables relevant to fall risk may still remain undiscovered. Future studies should incorporate a more comprehensive set of covariates, such as medication use, comorbidities, sensory or balance impairments, and psychosocial factors, to improve model accuracy and strengthen the evidence to better understand the biological, behavioral, and environmental contributors to fall risk.

Finally, the study sample may not be fully representative of the broader older adult population, as factors such as geographic location, socioeconomic status, and access to healthcare could influence both PA behaviors and fall incidents. Future research should explore populations with varying demographic and environmental backgrounds to determine whether these associations hold across different contexts.

## 5. Conclusions

In this study, we explore the complicated relationships between daily physical activity, elevated temperatures, sociodemographic characteristics, and fall incidents among low-income older adults. The results reveal that higher temperatures are associated with an increased incidence of falls, although engaging in more moderate-to-vigorous physical activity may serve as a protective factor against falls. The notable association between race and fall incidents underscores the necessity for further investigation into the disparities and access to fall prevention resources between different groups. Given that climate change is one of the most significant public health concerns [1–3], these findings highlight the importance of customizing fall prevention strategies to account for seasonal variations, as the risk of falls appears to increase during warmer months. Interventions should focus on promoting more intense physical activity as a form of protection while also considering environmental factors that may influence activity patterns and fall susceptibility. In addition, further research is essential for investigating the various contributing factors associated with fall risk, with an emphasis on identifying protective factors in individuals who remain fall-free despite shared demographic or environmental risks.

## Supplementary Material

Supplementary material

**Supplementary Materials:** The following supporting information can be downloaded at https://www.mdpi.com/article/10.3390/info16060442/s1, File S1: Supplementary Material: Effect of Elevated Temperature on Physical Activity and Falls in Low-Income Older Adults Using Zero-Inflated Poisson and Graphical Models.

## Figures and Tables

**Table 1.** Descriptive analysis of participant characteristics (*N* = 304).

Study Variables	Participants, *N* = 304

**Sociodemographic**	
Age (Years), mean (SD)	74.71 (7.23)
Gender	
Female	262 (86.2%)
Male	42 (13.8%)
Race/ethnicity	
Non-Hispanic Asian	22 (7.2%)
Non-Hispanic African American	117 (38.5%)
Hispanic	90 (29.6%)
Non-Hispanic White	69 (22.7%)
Education level	
High school or below	163 (53.6%)
College or higher	138 (45.8%)
Financial difficulty	
Adequate or less	64 (21.1%)
More than adequate	235 (77.3%)
Living condition	
Alone	168 (55.3%)
With others	133 (43.8%)
Self-rated health	
Excellent or very good	107 (35.2%)
Good or below	194 (63.8%)
**Physical activity: Accelerometer measurement**	
SB ^1^ (mins/day), mean (SD)	729.84 (112.88)
LPA ^2^ (mins/day), mean (SD)	204.70 (65.85)
MVPA ^3^ (mins/day), mean (SD)	31.35 (27.05)
**Fall events**	
None	264 (86.8%)
1	33 (10.9%)
2	6 (2.0%)
More than 2	1 (0.3%)

1 SB, sedentary behavior.

2 LPA, light-intensity physical activity.

3 MVPA, moderate-to-vigorous physical activity.

**Table 2.** Spearman correlation matrix of continuous variables.

	Temperature	SB	MVPA	Age	Fall events

Temperature	1.000				
SB	0.020	1.000			
MVPA	−0.008	−0.098 ***	1.000		
Age	0.018	0.089 ***	−0.304 ***	1.000	
Fall events	0.131 ***	−0.003	−0.036 **	0.015	1.000

** *p* < 0.01,

*** *p* < 0.001.

**Table 3.** Zero-inflated Poisson final model results.

	Dependent Variable = Fall Incidents		

Independent Variables	Estimate (*β*)	Standard Error	*p*-Value

**Intercept**	**−1.927**	**0.463**	**<0.0001**
**MVPA**	**−0.105**	**0.050**	**0.037**
**Temperature**	**0.550**	**0.047**	**<0.0001**
SB	0.023	0.048	0.636
Age	−0.006	0.006	0.312
Race/ethnicity			
Non-Hispanic African American	0.197	0.152	0.194
Hispanic	0.177	0.153	0.247
**Non-Hispanic White**	**0.839**	**0.152**	**<0.0001**
Financial difficulty			
More than adequate	0.149	0.095	0.116
MVPA × Temperature	−0.066	0.051	0.191
**Temperature × SB**	**−0.206**	**0.049**	**<0.0001**
**MVPA × SB**	**−0.169**	**0.048**	**0.0004**

**Table 4.** Weighted adjacency matrix of 11 features (nodes) in the Mixed Undirected Graphical Model (green: positive associations; red: negative associations; black: undefined sign; blank cells: no direct relationship between features).

	Temp	MVPA	SB	Fall	Age	Gendr	Race	Edu	Living	Financ

Temp										
MVPA	0.011									
SB	0.087	0.118								
Fall	0.263	0.081	0.073							
Age		0.286	0.117	0.031						
Gendr	0.091	0.037		0.163	0.093					
Race	0.121	0.174	0.084	0.460	0.220	0.596				
Edu	0.051	0.033		0.066	0.148	0.093	0.229			
Living	0.090		0.148		0.121	0.234	0.052	0.114		
Financ	0.034	0.150		0.205	0.266	0.116	0.142		0.041	
Health	0.095	0.069	0.031			0.363	0.433	0.315	0.126	0.158

Note: Variable names in this table align with node labels in Figure 3.

**Table 5.** Extended Bayesian Information Criterion values of each variable (node) for model selection in the Mixed Undirected Graphical Model.

Variables (Nodes)	EBIC Value

Temperature	748.5300
MVPA	788.4328
SB	826.1322
Fall events	716.3211
Age	784.1348
Gender	244.4354
Race/ethnicity	1197.0724
Education level	502.6574
Living condition	415.4221
Financial difficulty	945.5156
Self-rated health	402.2602

## Data Availability

The data presented in this study are available on request from the corresponding authors.

## Abbreviations

The following abbreviations are used in this manuscript:

AIC: Akaike Information Criterion
BIC: Bayesian Information Criterion
CI: Confidence interval
EBIC: Extended Bayesian Information Criterion
LOAs: Low-income older adults
LPA: Light-intensity physical activity
MVPA: Moderate-to-vigorous physical activity
MUGM: Mixed undirected graphical model
PA: Physical activity
RR: Relative risk
SB: Sedentary behavior
ZIP: Zero-inflated Poisson
