# Supplementary material for: Effect of Elevated Temperature on Physical Activity and Falls in Low-Income Older Adults Using Zero-Inflated Poisson and Graphical Models
Source: Information (Basel). Author manuscript; Available in PMC 2025 Aug 19. (PMC12360428; doi:10.3390/info16060442)

Supplementary Material: Effect of Elevated Temperature on Physical Activity and Falls in Low-Income Older Adults Using Zero-Inflated Poisson and Graphical Models

Tho Nguyen, Dahee Kim, Yingru Li, Christopher Emrich, Jennifer Crook, Ladda Thiamwong and Rui Xie

Load Libraries

```
library(dplyr)

##
## Attaching package: 'dplyr'

## The following objects are masked from 'package:stats':
##
##     filter, lag

## The following objects are masked from 'package:base':
##
##     intersect, setdiff, setequal, union

library(pscl)

## Warning: package 'pscl' was built under R version 4.4.2

## Classes and Methods for R originally developed in the
## Political Science Computational Laboratory
## Department of Political Science
## Stanford University (2002-2015),
## by and under the direction of Simon Jackman.
## hurdle and zeroinfl functions by Achim Zeileis.

library(mgm)

## Warning: package 'mgm' was built under R version 4.4.2

## This is mgm 1.2-14

## Please report issues on Github: https://github.com/jmbh/mgm/issues

library(qgraph)

## Warning: package 'qgraph' was built under R version 4.4.2

library(imputeTS)

## Warning: package 'imputeTS' was built under R version 4.4.2
```

```
## Registered S3 method overwritten by 'quantmod':
##   method            from
##   as.zoo.data.frame zoo
```

```
load("pa_temp_fall_1-24-25.RData")
```

1. ZIP regression

Normalize the variables

```
pa_temp_fall <- pa_temp_fall %>%
  mutate(mvpa_mean = scale(dur_day_total_MVPA_min),
         temp_mean = scale(avrg_temp),
         sb_mean = scale(dur_day_total_IN_min),
         gender_cat = as.factor(case_when(
           gender=="F" ~ 1,
           TRUE ~ 2)),
         race_cat = as.factor(case_when(
           race=="W" ~4,
           race=="H"~3,
           race=="AA"~2,
           TRUE ~1)),
         financial = as.factor(financial),
         education = as.factor(education),
         living = as.factor(living),
         health = as.factor(health))
```

Fit the ZIP model with interaction in the Poisson count part

```
zip_model <- zeroinfl(fall_inc ~ mvpa_mean + temp_mean + sb_mean + age +
                        gender_cat + race_cat + financial + living + health +
                        education + mvpa_mean:temp_mean + sb_mean:temp_mean +
                        mvpa_mean:sb_mean| 1,
                      data = pa_temp_fall,
                      dist = "poisson")
summary(zip_model)
```

```
##
## Call:
## zeroinfl(formula = fall_inc ~ mvpa_mean + temp_mean + sb_mean + age +
##     gender_cat + race_cat + financial + living + health + education +
##     mvpa_mean:temp_mean + sb_mean:temp_mean + mvpa_mean:sb_mean | 1,
##     data = pa_temp_fall, dist = "poisson")
##
## Pearson residuals:
##     Min      1Q  Median      3Q     Max
## -0.7208 -0.3618 -0.2958 -0.2222 11.4579
##
## Count model coefficients (poisson with log link):
##                      Estimate Std. Error z value Pr(>|z|)
## (Intercept)         -1.950022   0.471487  -4.136 3.54e-05 ***
```

```
## mvpa_mean           -0.103331   0.050551   -2.044 0.040946 *
## temp_mean            0.552493   0.047534   11.623  < 2e-16 ***
## sb_mean              0.024125   0.048056    0.502 0.615655
## age                 -0.005317   0.005676   -0.937 0.348876
## gender_cat2         -0.108277   0.111582   -0.970 0.331858
## race_cat2            0.164586   0.154525    1.065 0.286826
## race_cat3            0.148700   0.155006    0.959 0.337400
## race_cat4            0.808962   0.156674    5.163 2.43e-07 ***
## financial1           0.147257   0.094812    1.553 0.120386
## living2              0.009538   0.075039    0.127 0.898858
## health1              0.017701   0.078053    0.227 0.820588
## education1           0.058829   0.075313    0.781 0.434728
## mvpa_mean:temp_mean -0.067600   0.050681   -1.334 0.182256
## temp_mean:sb_mean   -0.207251   0.049166   -4.215 2.49e-05 ***
## mvpa_mean:sb_mean   -0.169895   0.047763   -3.557 0.000375 ***
##
## Zero-inflation model coefficients (binomial with logit link):
##             Estimate Std. Error z value Pr(>|z|)
## (Intercept)  -0.6805     0.2864  -2.376   0.0175 *
## ---
## Signif. codes:  0 '***' 0.001 '**' 0.01 '*' 0.05 '.' 0.1 ' ' 1
##
## Number of iterations in BFGS optimization: 28
## Log-likelihood: -2521 on 17 Df
```

Check the model diagnostics

```
cat("Log-likelihood:", logLik(zip_model), "\n")

## Log-likelihood: -2521.062

cat("AIC:", AIC(zip_model), "\n")

## AIC: 5076.123

cat("BIC:", BIC(zip_model), "\n")

## BIC: 5192.848
```

Backward model selection

```
zip2 <- zeroinfl(fall_inc ~ mvpa_mean + temp_mean + sb_mean + age +
gender_cat +
                   race_cat + financial + health + education +
                   mvpa_mean:temp_mean + sb_mean:temp_mean +
                   mvpa_mean:sb_mean | 1,
                 data = pa_temp_fall,
                 dist = "poisson")
summary(zip2)

##
## Call:
```

```
## zeroinfl(formula = fall_inc ~ mvpa_mean + temp_mean + sb_mean + age +
##     gender_cat + race_cat + financial + health + education +
mvpa_mean:temp_mean +
##     sb_mean:temp_mean + mvpa_mean:sb_mean | 1, data = pa_temp_fall, dist =
"poisson")
##
## Pearson residuals:
##     Min      1Q  Median      3Q     Max
## -0.7219 -0.3617 -0.2956 -0.2220 11.4913
##
## Count model coefficients (poisson with log link):
##                      Estimate Std. Error z value Pr(>|z|)
## (Intercept)         -1.942227   0.467418  -4.155 3.25e-05 ***
## mvpa_mean           -0.103512   0.050529  -2.049 0.040506 *
## temp_mean            0.552452   0.047525  11.624  < 2e-16 ***
## sb_mean              0.024314   0.048029   0.506 0.612697
## age                 -0.005386   0.005650  -0.953 0.340434
## gender_cat2         -0.105936   0.110042  -0.963 0.335706
## race_cat2            0.165369   0.154406   1.071 0.284169
## race_cat3            0.148639   0.154995   0.959 0.337561
## race_cat4            0.810041   0.156447   5.178 2.25e-07 ***
## financial1           0.147835   0.094698   1.561 0.118494
## health1              0.017176   0.077933   0.220 0.825567
## education1           0.058369   0.075225   0.776 0.437793
## mvpa_mean:temp_mean -0.067632   0.050677  -1.335 0.182017
## temp_mean:sb_mean   -0.207239   0.049163  -4.215 2.49e-05 ***
## mvpa_mean:sb_mean   -0.169889   0.047755  -3.557 0.000374 ***
##
## Zero-inflation model coefficients (binomial with logit link):
##             Estimate Std. Error z value Pr(>|z|)
## (Intercept)  -0.6818     0.2865   -2.38   0.0173 *
## ---
## Signif. codes:  0 '***' 0.001 '**' 0.01 '*' 0.05 '.' 0.1 ' ' 1
##
## Number of iterations in BFGS optimization: 27
## Log-likelihood: -2521 on 16 Df
```

```
zip3 <- zeroinfl(fall_inc ~ mvpa_mean + temp_mean + sb_mean + age +
gender_cat +
                   race_cat + financial + education + mvpa_mean:temp_mean +
                   sb_mean:temp_mean + mvpa_mean:sb_mean| 1,
                 data = pa_temp_fall,
                 dist = "poisson")
summary(zip3)
```

```
##
## Call:
## zeroinfl(formula = fall_inc ~ mvpa_mean + temp_mean + sb_mean + age +
##     gender_cat + race_cat + financial + education + mvpa_mean:temp_mean +
##     sb_mean:temp_mean + mvpa_mean:sb_mean | 1, data = pa_temp_fall, dist =
```

```
"poisson")
##
## Pearson residuals:
##     Min      1Q  Median      3Q     Max
## -0.7244 -0.3614 -0.2959 -0.2219 11.4451
##
## Count model coefficients (poisson with log link):
##                      Estimate Std. Error z value Pr(>|z|)
## (Intercept)         -1.941935   0.467580  -4.153 3.28e-05 ***
## mvpa_mean           -0.102586   0.050345  -2.038 0.041583 *
## temp_mean            0.552905   0.047479  11.645  < 2e-16 ***
## sb_mean              0.024101   0.048011   0.502 0.615667
## age                 -0.005373   0.005653  -0.950 0.341874
## gender_cat2         -0.103200   0.109314  -0.944 0.345135
## race_cat2            0.168349   0.153797   1.095 0.273684
## race_cat3            0.151932   0.154257   0.985 0.324660
## race_cat4            0.814898   0.154880   5.261 1.43e-07 ***
## financial1           0.148316   0.094686   1.566 0.117257
## education1           0.060618   0.074518   0.813 0.415954
## mvpa_mean:temp_mean -0.067642   0.050641  -1.336 0.181645
## temp_mean:sb_mean   -0.207100   0.049156  -4.213 2.52e-05 ***
## mvpa_mean:sb_mean   -0.169338   0.047671  -3.552 0.000382 ***
##
## Zero-inflation model coefficients (binomial with logit link):
##             Estimate Std. Error z value Pr(>|z|)
## (Intercept)  -0.6811     0.2863  -2.379   0.0174 *
## ---
## Signif. codes:  0 '***' 0.001 '**' 0.01 '*' 0.05 '.' 0.1 ' ' 1
##
## Number of iterations in BFGS optimization: 27
## Log-likelihood: -2521 on 15 Df

zip4 <- zeroinfl(fall_inc ~ mvpa_mean + temp_mean + sb_mean + age +
gender_cat +
                   race_cat + financial + mvpa_mean:temp_mean +
                   sb_mean:temp_mean + mvpa_mean:sb_mean| 1,
                 data = pa_temp_fall,
                 dist = "poisson")
summary(zip4)

##
## Call:
## zeroinfl(formula = fall_inc ~ mvpa_mean + temp_mean + sb_mean + age +
##     gender_cat + race_cat + financial + mvpa_mean:temp_mean +
sb_mean:temp_mean +
##     mvpa_mean:sb_mean | 1, data = pa_temp_fall, dist = "poisson")
##
## Pearson residuals:
##     Min      1Q  Median      3Q     Max
## -0.7173 -0.3622 -0.2958 -0.2222 11.5835
```

```
##
## Count model coefficients (poisson with log link):
##                        Estimate Std. Error z value Pr(>|z|)    
## (Intercept)           -1.896586   0.463979  -4.088 4.36e-05 ***
## mvpa_mean             -0.102895   0.050287  -2.046 0.040740 *  
## temp_mean              0.551017   0.047394  11.626  < 2e-16 ***
## sb_mean                0.023837   0.047956   0.497 0.619150    
## age                   -0.005766   0.005630  -1.024 0.305757    
## gender_cat2           -0.101034   0.109283  -0.925 0.355217    
## race_cat2              0.178690   0.153248   1.166 0.243607    
## race_cat3              0.160636   0.153912   1.044 0.296632    
## race_cat4              0.837206   0.152473   5.491 4.00e-08 ***
## financial1             0.150533   0.094696   1.590 0.111916    
## mvpa_mean:temp_mean   -0.066838   0.050505  -1.323 0.185706    
## temp_mean:sb_mean     -0.206192   0.049105  -4.199 2.68e-05 ***
## mvpa_mean:sb_mean     -0.167840   0.047578  -3.528 0.000419 ***
##
## Zero-inflation model coefficients (binomial with logit link):
##             Estimate Std. Error z value Pr(>|z|)  
## (Intercept)  -0.6794     0.2860  -2.376   0.0175 *
## ---
## Signif. codes:  0 '***' 0.001 '**' 0.01 '*' 0.05 '.' 0.1 ' ' 1
##
## Number of iterations in BFGS optimization: 25
## Log-likelihood: -2521 on 14 Df
```

```
zip5 <- zeroinfl(fall_inc ~ mvpa_mean + temp_mean + sb_mean + age + race_cat
+
                   financial + mvpa_mean:temp_mean + sb_mean:temp_mean +
                   mvpa_mean:sb_mean| 1,
                 data = pa_temp_fall,
                 dist = "poisson")
summary(zip5)
```

```
##
## Call:
## zeroinfl(formula = fall_inc ~ mvpa_mean + temp_mean + sb_mean + age +
##     race_cat + financial + mvpa_mean:temp_mean + sb_mean:temp_mean +
##     mvpa_mean:sb_mean | 1, data = pa_temp_fall, dist = "poisson")
##
## Pearson residuals:
##     Min      1Q  Median      3Q     Max
## -0.7108 -0.3616 -0.2959 -0.2223 11.5981
##
## Count model coefficients (poisson with log link):
##                        Estimate Std. Error z value Pr(>|z|)    
## (Intercept)           -1.927494   0.462677  -4.166 3.10e-05 ***
## mvpa_mean             -0.104580   0.050223  -2.082 0.037314 *  
## temp_mean              0.550119   0.047389  11.609  < 2e-16 ***
## sb_mean                0.022692   0.047905   0.474 0.635717    
```

```
## age                 -0.005684   0.005623  -1.011 0.312084
## race_cat2            0.197443   0.151994   1.299 0.193939
## race_cat3            0.177175   0.152951   1.158 0.246710
## race_cat4            0.839069   0.152431   5.505 3.70e-08 ***
## financial1           0.148784   0.094673   1.572 0.116055
## mvpa_mean:temp_mean -0.066061   0.050527  -1.307 0.191062
## temp_mean:sb_mean   -0.205657   0.049073  -4.191 2.78e-05 ***
## mvpa_mean:sb_mean   -0.168537   0.047543  -3.545 0.000393 ***
##
## Zero-inflation model coefficients (binomial with logit link):
##             Estimate Std. Error z value Pr(>|z|)
## (Intercept)  -0.6817     0.2865  -2.379   0.0174 *
## ---
## Signif. codes:  0 '***' 0.001 '**' 0.01 '*' 0.05 '.' 0.1 ' ' 1
##
## Number of iterations in BFGS optimization: 24
## Log-likelihood: -2522 on 13 Df
```

Compare models using AIC and BIC

```
AIC(zip_model, zip2, zip3, zip4, zip5)
```

```
##           df      AIC
## zip_model 17 5076.123
## zip2      16 5074.139
## zip3      15 5072.188
## zip4      14 5070.849
## zip5      13 5069.720
```

```
BIC(zip_model, zip2, zip3, zip4, zip5)
```

```
##           df      BIC
## zip_model 17 5192.848
## zip2      16 5183.998
## zip3      15 5175.180
## zip4      14 5166.975
## zip5      13 5158.980
```

2. Mixed Undirected Graphical Model

Prepare the data

```
temp = ts(data = pa_temp_fall$avrg_temp)

age = ts(data = pa_temp_fall$age)
age_imputed <- na_kalman(age) #impute missing values by kalman smoothing

gender = ts(data=pa_temp_fall$gender_cat)
race = ts(data=pa_temp_fall$race_cat)
```

```
edu = ts(data=pa_temp_fall$education)
living = ts(data=pa_temp_fall$living)
finance = ts(data=pa_temp_fall$financial)
health = ts(data=pa_temp_fall$health)

mvpa = ts(data = pa_temp_fall$dur_day_total_MVPA_min)
sb = ts(data = pa_temp_fall$dur_day_total_IN_min)

fall = ts(data = pa_temp_fall$fall_inc)
```

Create the data matrix

```
data = cbind(temp,
mvpa,sb,fall,age_imputed,gender,race,edu,living,finance,health)

data_matrix =  matrix(data,ncol=11)
colnames(data_matrix) <- c("Temp", "MVPA", "SB", "Fall","Age","Gendr","Race",
"Edu", "Living", "Financ", "Health")
type <- c("g", "g", "g", "p","g", "c", "c", "c", "c", "c", "c")
levels <- c(1, 1, 1, 1, 1, 2, 4, 2, 2, 2, 2)
```

Calculate weights

```
n_total <- length(fall)
n_class_0 <- sum(fall == 0)
n_class_1 <- sum(fall == 1)
n_class_2 <- sum(fall == 2)

weight_0 <- n_total / (2 * n_class_0)
weight_1 <- n_total / (2 * n_class_1)
weight_2 <- n_total / (2 * n_class_2)

weights <- ifelse(fall == 0, weight_0, ifelse(fall==1, weight_1, weight_2))
standardized_weights <- weights / sum(weights)
```

Fit the MUGM model

```
graph <- mgm(data_matrix, type = type,level=levels, k=2,
             weights=standardized_weights, scale = TRUE, signInfo = TRUE,
             lambdaSel="CV", lambdaFolds=10, overparameterize = F, ruleReg =
"AND")

##   |
|                                                                      |   0%
|
|------                                                                |   9%
|
|-------------                                                         |  18%
|
|-------------------                                                   |  27%
|
```

```
|-------------------------                                                    |  36%
|
|--------------------------------                                             |  45%
|
|--------------------------------------                                       |  55%
|
|---------------------------------------------                                |  64%
|
|---------------------------------------------------                          |  73%
|
|---------------------------------------------------------                    |  82%
|
|----------------------------------------------------------------             |  91%
|
|-----------------------------------------------------------------------------| 100%
## Note that the sign of parameter estimates is stored separately; see ?mgm
```

Weighted adjacency matrix

```
graph$pairwise$wadj

##             [,1]       [,2]       [,3]       [,4]       [,5]       [,6]
##  [1,] 0.00000000 0.01088840 0.09122236 0.26163997 0.00000000 0.08663715
##  [2,] 0.01088840 0.00000000 0.11946879 0.08005499 0.27657299 0.03523504
##  [3,] 0.09122236 0.11946879 0.00000000 0.07666995 0.11030526 0.01630478
##  [4,] 0.26163997 0.08005499 0.07666995 0.00000000 0.00000000 0.15607296
##  [5,] 0.00000000 0.27657299 0.11030526 0.00000000 0.00000000 0.04918612
##  [6,] 0.08663715 0.03523504 0.01630478 0.15607296 0.04918612 0.00000000
##  [7,] 0.13167703 0.17882346 0.08828617 0.43431852 0.17654740 0.59760517
##  [8,] 0.05284258 0.03453650 0.00000000 0.06565777 0.13030500 0.08993898
##  [9,] 0.08173001 0.00000000 0.14545918 0.00000000 0.08426692 0.20368175
## [10,] 0.00000000 0.14791025 0.00000000 0.20045796 0.23033847 0.10463176
## [11,] 0.09757517 0.06995489 0.03846319 0.00000000 0.00000000 0.36129310
##             [,7]       [,8]       [,9]     [,10]      [,11]
##  [1,] 0.13167703 0.05284258 0.08173001 0.0000000 0.09757517
##  [2,] 0.17882346 0.03453650 0.00000000 0.1479103 0.06995489
##  [3,] 0.08828617 0.00000000 0.14545918 0.0000000 0.03846319
##  [4,] 0.43431852 0.06565777 0.00000000 0.2004580 0.00000000
##  [5,] 0.17654740 0.13030500 0.08426692 0.2303385 0.00000000
##  [6,] 0.59760517 0.08993898 0.20368175 0.1046318 0.36129310
##  [7,] 0.00000000 0.24044335 0.04816878 0.1552366 0.48514680
##  [8,] 0.24044335 0.00000000 0.09960576 0.0000000 0.31923728
##  [9,] 0.04816878 0.09960576 0.00000000 0.0000000 0.11141936
## [10,] 0.15523665 0.00000000 0.00000000 0.0000000 0.15927668
## [11,] 0.48514680 0.31923728 0.11141936 0.1592767 0.00000000

graph$pairwise$signs

##       [,1] [,2] [,3] [,4] [,5] [,6] [,7] [,8] [,9] [,10] [,11]
##  [1,]   NA    1   -1    1   NA    0    0    0    0    NA     0
##  [2,]    1   NA   -1   -1   -1    0    0    0   NA     0     0
```

```
## [3,]  -1  -1  NA   1   1   0   0  NA   0   NA   0
## [4,]   1  -1   1  NA  NA   0   0   0  NA    0  NA
## [5,]  NA  -1   1  NA  NA   0   0   0   0    0  NA
## [6,]   0   0   0   0   0  NA   0   0   0    0   0
## [7,]   0   0   0   0   0   0  NA   0   0    0   0
## [8,]   0   0  NA   0   0   0   0  NA   0   NA   0
## [9,]   0  NA   0  NA   0   0   0   0  NA   NA   0
## [10,] NA   0  NA   0   0   0   0  NA  NA   NA   0
## [11,]  0   0   0  NA  NA   0   0   0   0    0  NA
```

Check the EBIC values

```
ebic_values <- data.frame(Node = 1:11, EBIC = sapply(graph$nodemodels,
function(x) x$EBIC))
print(ebic_values)

##    Node      EBIC
## 1     1  748.5300
## 2     2  788.4328
## 3     3  826.1322
## 4     4  716.3211
## 5     5  784.1348
## 6     6  244.4354
## 7     7 1197.0724
## 8     8  502.6574
## 9     9  415.4221
## 10   10  945.5156
## 11   11  402.2602
```

Check the number of non-zero edges

```
non_zero_count <- sum(graph$pairwise$wadjNodewise != 0)
total_count <- length(graph$pairwise$wadjNodewise)
(proportion_non_zero <- non_zero_count / total_count)

## [1] 0.7933884
```

Visualize the graph

```
cat <- c(1,2,3,4,5,6,7,8,9,10,11)

# Define custom colors for each node (matching the number of nodes in the
graph)
node_colors <- rep("lightblue", ncol(data_matrix))  # Default color for all
node_colors[c(2,3)] <- "#fdb462"
node_colors[c(1)] <- "#386cb0"
node_colors[c(4)] <- "#fb9a99"



# Plot the graph with custom node colors
g1 = qgraph(graph$pairwise$wadj,
            edge.color = graph$pairwise$edgecolor,
```

```
             layout = "circle",
             groups = cat,
             palette = "pastel",
             labels = colnames(data_matrix),
             theme = "TeamFortress",
             vsize = 10,
             color = node_colors)
```

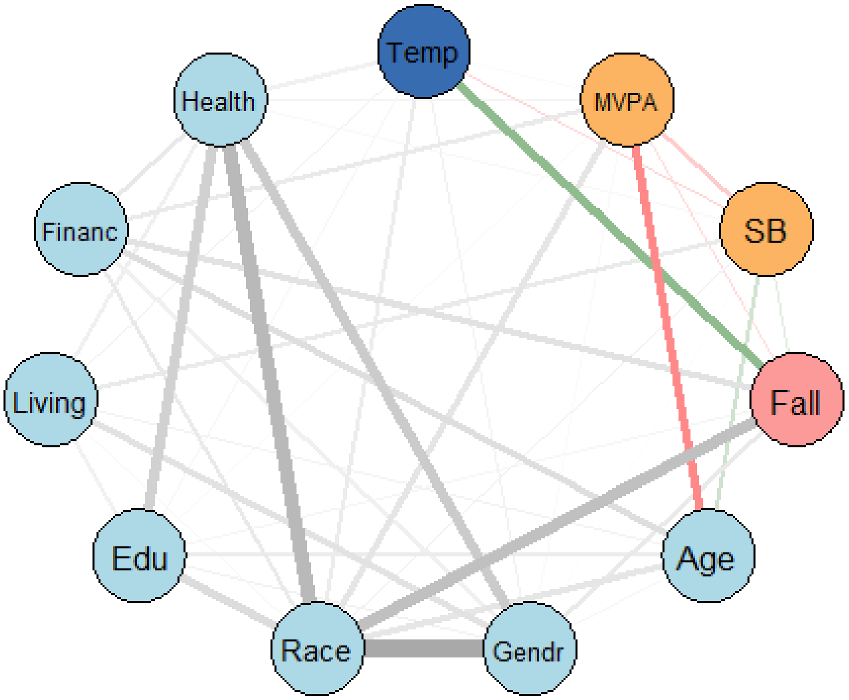

Supplement: Supplementary material [file NIHMS2096981-supplement-Supplementary_material.pdf]
